# Organophosphate Toxicity Presenting as a Stroke Alert

**DOI:** 10.7759/cureus.36882

**Published:** 2023-03-29

**Authors:** Martin J Morales-Cruz, Bridget Scheveck, Vanessa I Diaz, Keegan S McNally, Jennifer Elfman, Latha Ganti

**Affiliations:** 1 Emergency Medicine, HCA Florida Osceola Hospital, Kissimmee, USA; 2 Emergency Medicine, Brown University, Providence, USA; 3 Emergency Medicine, Envision Physician Services, Plantation, USA; 4 Emergency Medicine, University of Central Florida College of Medicine, Orlando, USA

**Keywords:** malathion, toxicology, altered mental status, stroke, organophosphate pesticide poisoning

## Abstract

Altered mental status is a common emergency department presentation. It has a broad differential and can be particularly challenging when the patient is unable to give a history and collateral information is not immediately available. The authors present a case of altered mental status initially brought in as a stroke alert but later discovered to be intentional organophosphate ingestion. Although organophosphate poisoning is relatively rare in the United States, it should be considered in patients with altered mental status with miosis who are unresponsive to naloxone, especially in the setting of bradycardia or copious secretions.

## Introduction

Organophosphates are used as pesticides around the world, in both agriculture and sprays intended for home use. With pesticides widely available globally, it is estimated that 3,000,000 exposures occur annually to organophosphate or carbamate agents. Up to 300,000 deaths are estimated globally per year from these agents [1]. In the United States, a 2008 report from the National Poison Data System reported more than 8000 cases of exposure leading to fewer than 15 deaths [2]. The authors report a case of intentional ingestion of home-use organophosphate pesticide spray presenting as a stroke alert due to an initially undifferentiated altered mental status.

## Case presentation

An originally unidentified 76-year-old male with unknown past medical history was brought in by emergency medical services (EMS) after being found unresponsive in his car. Per the EMS report, police were called after the patient was seen in front of a fire station stumbling, vomiting, defecating, and urinating on the road. Police officers arrived, and the patient drove off in his vehicle. Shortly thereafter, he was found behind the fire station, inside his car, unresponsive. EMS denied seeing any drugs, drug paraphernalia, or alcohol in the vehicle. He was taken out of his car by EMS staff and became combative en route.

**Figure 1:**
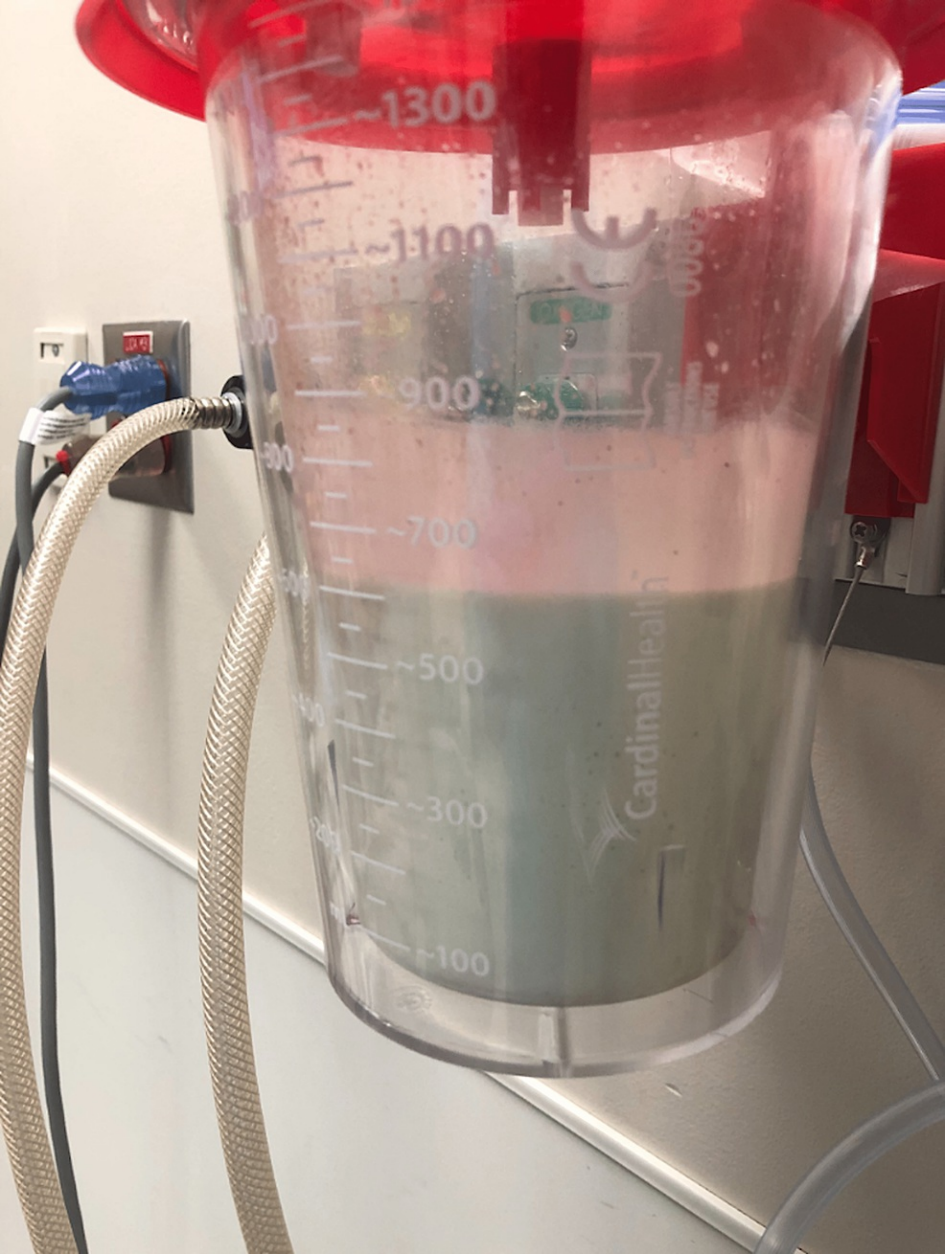
Contents of patient's orogastric tube

Upon arrival at the emergency room, the patient was stroke-alerted for altered mental status. His initial blood pressure was 190/83 mmHg, his heart rate was 94 beats per minute, his respiratory rate was 18 breaths per minute, his oxygen saturation was 98% on room air, the temperature was 37.0 degrees Celsius, and point-of-care blood glucose obtained was in the 100s. The initial National Institutes of Health Stroke Scale (NIHSS) score was 31. The Glasgow Coma Scale (GCS) was 3. Pupils were noted to be pinpoint; therefore, he was administered IV naloxone 4 mg without any significant improvement. He was emergently intubated for airway protection and taken for computed tomography (CT). The CT of the brain was negative for any acute intracranial pathology. The last known well time was unknown; therefore, he was not a candidate for IV alteplase. There was no large vessel occlusion on CT angiography (CTA), thus he was not a candidate for thrombectomy either. Lactic acid was elevated at 6.9; the patient received a sepsis workup and was placed on maintenance fluids. An orogastric tube was placed, which yielded copious white secretions (Figure 1).

**Figure 2:**
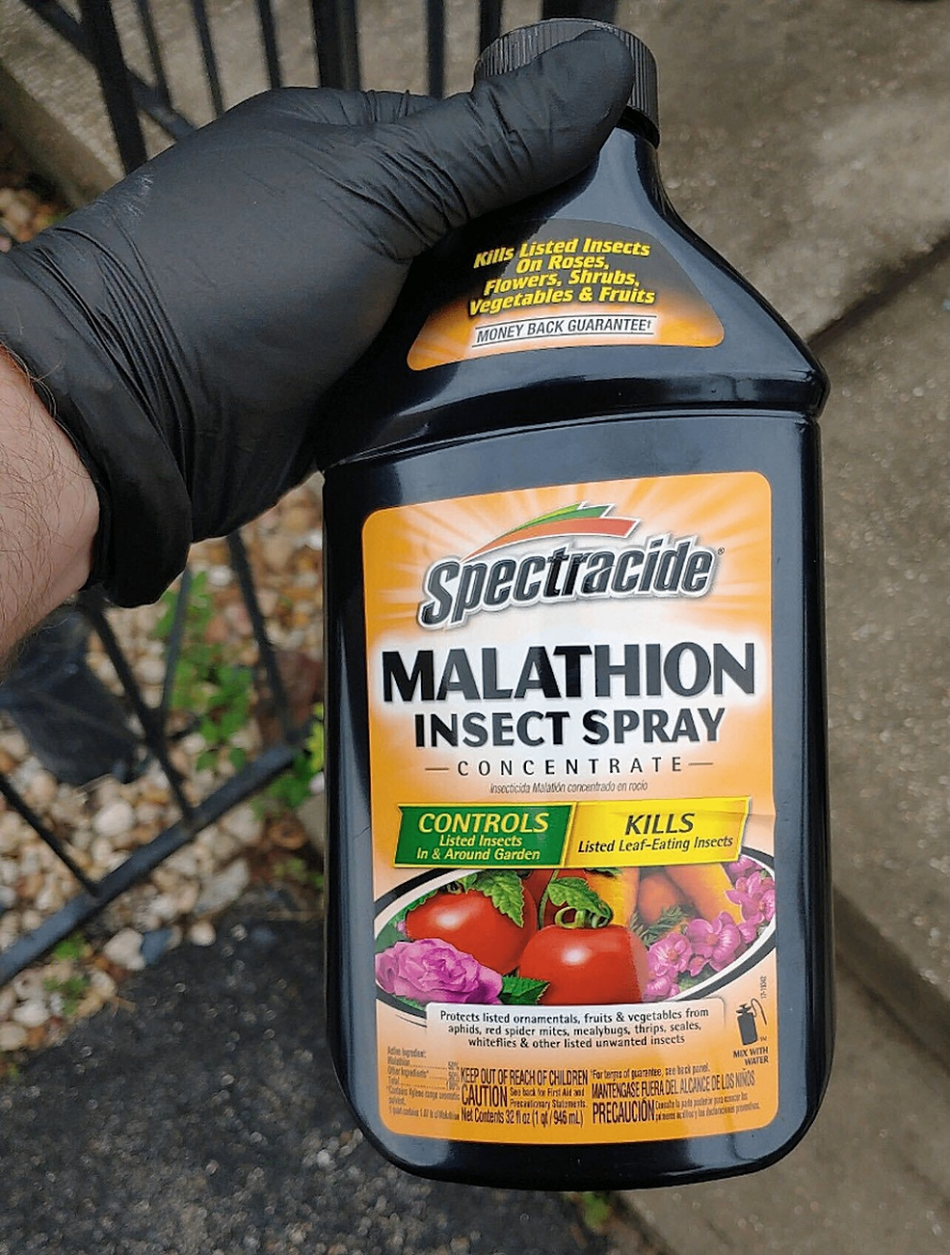
Insecticide ingested by the patient

The patient was seen by the intensive care day team in the morning, intubated, and sedated. The patient was noted to be drooling, having pinpoint pupils bilaterally, and being bradycardic. The patient was identified overnight, and the family presented at the bedside. They reported the patient was talking about death all morning and what they should do once he was dead before disappearing around noon. The family reported the patient was drinking alcohol, and they found a bag with a bottle of insecticide near where he was last seen. The family was concerned that the patient may have intentionally drunk the spray. Malathion is a common ingredient found in several insecticides and is classified as an organophosphate. The insecticide was Spectracide® Malathion Insect Spray (Figure 2).

Poison control was contacted immediately after suspicion arose of organophosphate poisoning. Atropine and pralidoxime (2-PAM) were given with a good response. Secretions and bradycardia improved, and miosis began to improve. However, a few hours later, the patient became symptomatic again and required multiple additional doses of atropine. At the recommendation of poison control, the patient was started on a continuous 2-PAM infusion for 12 hours. The patient stabilized on infusion and did not require additional doses of atropine overnight. The following day, after the 2-PAM infusion finished, the patient developed episodes of bradycardia with associated mild hypotension responsive to atropine. The patient gradually required less and less frequent dosing of atropine as he continued to clinically improve.

The patient was extubated on hospital day five. The patient was initially guarded about organophosphate poisoning but eventually did report that he drank "three mouthfuls of poison" in an attempt to kill himself. The patient was transferred to a step-down level of care on hospital day six and discharged on hospital day 11 after psychiatric treatment was established and the psychiatric hold was lifted by the in-patient psychiatry team.

## Discussion

Malathion is a common ingredient found in thousands of insecticides available for sale within the United States. Malathion works by acting as an acetylcholinesterase inhibitor, thus leading to overstimulation of the nicotinic and muscarinic acetylcholine receptors in the CNS, autonomic, and neuromuscular systems. Individuals may be exposed via inhalation, oral ingestion, or skin absorption [3]. Ingestion can lead to the classic symptoms of SLUDGEM: salivation, lacrimation, urination, defecation, gastrointestinal motility, emesis, and miosis. The diagnosis of organophosphate poisoning is typically made on the basis of clinical suspicion. One of the most important initial steps prior to patient examination and treatment involves decontamination. It is vital to remove any remaining substrate on the patient's body to prevent inadvertent exposure to all providers.

The primary treatment for organophosphate ingestion includes atropine, 2-PAM, and benzodiazepine administration. Even with appropriate and early care, the fatality rate remains around 15% [4]. One of the main concerns due to organophosphate poisoning is a respiratory compromise due to excessive airway secretions. Atropine is a muscarinic antagonist and is administered to dry secretions to assist with adequate oxygen control. Atropine is also administered to improve cardiac function, combat bradycardia, and increase the patient’s heart rate. The dosage of atropine varies widely but is typically 1mg to 5mg and can be administered as needed every three to five minutes [5].

The other primary treatment is 2-PAM. Acetylcholinesterase receptors are inactivated by phosphorylation due to exposure to the organophosphate. The 2-PAM re-activates the receptors by removing the phosphoryl group from the active site [4]. 2-PAM is used in conjunction with atropine; atropine should show some effectiveness prior to the administration of 2-PAM [1,3]. The response to 2-PAM is highly variable and also depends on the time since exposure, the amount of organophosphate, as well as the type. Inhibited acetylcholinesterase receptors age through the loss of attached alkyl groups bound to the attached phosphate [4]. Once the receptor has aged, it cannot be reversed. The rate of aging varies by the type of organophosphate. The World Health Organization currently recommends that all patients requiring atropine also receive an oxime such as 2-PAM. The bolus dose of 2-PAM is typically 20 to 40 mg/kg (1 gram to 2 grams) with a maximum dose of 2 grams [5].

Patients with organophosphate poisoning can also develop agitated delirium for a number of different reasons, such as co-ingested alcohol, hypoxemia, or the organophosphate itself. Acutely agitated patients will benefit from treatment with a benzodiazepine, such as diazepam [4,5]. Benzodiazepines can also be used to treat patients who have seizures, although seizures are rare in patients who are well-oxygenated after exposure to pesticide poisoning. Some animal studies have shown diazepam to prevent neural damage and respiratory failure; therefore, the World Health Organization recommends diazepam as the benzodiazepine of choice [5].

Magnesium sulfate (MgSO4) is another medication that is currently being investigated for organophosphate poisoning. It is thought that magnesium inhibits acetylcholine release in the central nervous system (CNS) and peripheral sympathetic and parasympathetic nervous systems by blocking calcium channels [6]. Magnesium is thought to reduce arrhythmias and decrease CNS overstimulation [5]. In small studies, it was suggested to improve neuromuscular function, reduce mortality, decrease therapy costs, and decrease hospitalization days. There was a trend toward reduced mortality with larger magnesium doses (4 g, 8 g, and 12 g versus 16 g); however, larger randomized trials are needed to confirm [6,7].

Calcium channel blockers (CCB) have also been used to treat organophosphate poisoning (OP) and work in the same way as MgSO4, blocking calcium channels and subsequently decreasing acetylcholine release in the neuromuscular junction. CCBs have been found to be associated with fewer pathological electrocardiographic changes as well as having a protective effect on myocardial injury caused by OP. A recent systematic review of both pre-clinical and clinical studies, including four randomized controlled trials, studied the efficacy of both MgSO4 and CCB in acute organophosphate poisoning [8]. The authors concluded that, although the current evidence is limited and, as of now, not enough to recommend their use in clinical practice, MgSO4 and CCB represent promising adjunct treatments in acute OP. It is clear that more abundant evidence, particularly found in large-scale comparative clinical trials, is required to recommend the routine use of MgS04 and CCB in organophosphate poisoning.

## Conclusions

Although toxidromes can be difficult to identify without a history suggesting exposure, they are an important consideration in the undifferentiated, altered patient. This case also demonstrated the importance of obtaining collateral histories from family members or other witnesses whenever possible. Once organophosphate poisoning is identified, the mainstay of treatment is atropine, 2-PAM, and benzodiazepines. Poison Control and hospital pharmacists are vital resources in the management of this toxidrome and a multidisciplinary approach should be utilized to care for critically ill patients.
